# Small worms big discoveries: *Galleria mellonella* as a model for *Campylobacter jejuni* infection

**DOI:** 10.3389/fcimb.2025.1686074

**Published:** 2025-09-30

**Authors:** Todd P. Primm, Samuel R. Cannon, Anand B. Karki

**Affiliations:** Department of Biological Sciences, Sam Houston State University, Huntsville, TX, United States

**Keywords:** *Campylobacter jejuni*, *Galleria mellonella*, virulence, infection model, capsule

## Abstract

*Galleria mellonella* larva have served as a simple, cost-effective model for studying innate immunity and *Campylobacter jejuni* infection. The model commonly employs an acute, high-dose septic infection via hemocoel injection, with observable endpoints of death and melanization. Studies using *G. mellonella* have provided insights into *C. jejuni* virulence factors, including the capsule, transcriptional regulators, outer membrane vesicles, and a T6SS. It has revealed signals for virulence, such as pancreatic amylase and growth temperature, and also allowed for comparisons between *C. jejuni* strains and across multiple species in the genus. Limitations include the use of high bacterial doses that may obscure the role of specific virulence factors, lack of accounting for larval size variations, and unclear connection to the human anaerobic, microbially-rich gut environment. Future development of this model could allow oral infections for exploring pathogen-microbiome interactions and further assessing mechanisms of this important pathogen.

## Introduction

*Campylobacter* is the leading cause of bacterial foodborne gastroenteritis worldwide. While there are 50 valid species in this genus of Gram-negative curved rods (Parte et al., 2020), *C. jejuni* and *C. coli* are responsible for more than 90% of human infections (Tikhomirova et al., 2024). Typically, humans have self-limiting gastroenteritis, with a fever and vomiting phase of one to three days, then abdominal pain with watery or bloody diarrhea for an additional three to seven days (Kim et al., 2021). Antibiotic treatment is uncommon, reserved for immunocompromised patients, and typically straightforward, although fluoroquinolone resistance is increasing (Sproston et al., 2018; Whelan et al., 2019). However, rare but chronic post-infection complications can occur, including Guillain-Barre syndrome and reactive arthritis. These complications are likely due to antigen cross-reactivity (Finsterer, 2022) between human antigens and *C. jejuni* unusual glycomodifications on the capsular polysaccharides, lipooligosaccharides (LOS), and flagella (Omole et al., 2024). There are two subspecies within *C. jejuni*, which are *Cj* subsp. *jejuni* and *Cj* subsp. *doylei*, which is rare and linked to human cases of gastroenteritis that escape the gut and lead to bacteremia (Miller et al., 2007). This review will focus on *Cj* subsp. *jejuni*, as none of the 24 studies discussed here included *Cj* subsp. *doylei*. *Campylobacter* has some major differences compared to other common enterics, such as *Salmonella* or *Enterobacter*. It is microaerobic (preferring ~5% O_2_), has a comparatively small genome, different virulence factors, and a relatively low infective dose.

Many virulence models have been developed for *C. jejuni*, none being a close match to the human clinical presentation. The models include human intestinal epithelial cells in culture (for adhesion and invasion) (Harrer et al., 2019), ferrets (Nemelka et al., 2009), infant rabbits (Shang et al., 2016), pigs (Rath et al., 2022), *Drosophila* (Myles et al., 2024), antibiotic pre-treated mice (Yu et al., 2016), primates (Crofts et al., 2018), *Acanthamoeba* (Shagieva et al., 2021), and in limited studies even human volunteers (Crofts et al., 2018). Since chickens are a major reservoir and source for human infections, chick colonization (Davis and DiRita, 2008) is also an important system, although not modeling human infections.

## *Galleria* as a model

Larva of *Galleria mellonella*, the greater wax moth or the honeycomb moth, have been used as a simple, inexpensive model for interactions of innate host defense with pathogens (Wojda et al., 2020). Studies with 11 different bacterial pathogens, including *C. jejuni*, are summarized in a 2021 review (Ménard et al., 2021). Immune defenses in the body cavity include antimicrobial peptides, lysozyme, six types of hemocytes, and melanization. Methods typically involve infection of the larva with death as an endpoint. The immune reaction of melanization is also observable, as it is easily visualized as darkening. Melanization can be recorded in more detail by pattern (Hesketh-Best et al., 2021) or in a scoring system (Mehat et al., 2018; Emery et al., 2021; Ménard et al., 2021). Hemolymph can be isolated by squeezing from the larva’s body, then used to microscopically examine bacterial presence in fluids or hemocytes. Tissues can be isolated and examined similarly. In addition to pathogenesis, larva have been used to examine antimicrobials, probiotics, and phage therapy (Ménard et al., 2021). *G. mellonella* are one of the few insect models that allow for incubation at mammalian body temperature of 37°C. With developments in genetic manipulation, genomics, and microbiomes, this system will continue to expand in utility for infection biology. Gene knockdown is already available (Dutta et al., 2021). No review of the *Galleria* model with *Campylobacter* has been published, although a book chapter in 2017 focused on methods did include a brief evaluation of the field (Askoura and Stintzi, 2017).

## Virulence factors

The earliest publication using *Galleria* with *Campylobacter* was in 2010 (Champion et al., 2010), which shaped the field as far as experimental methods and approaches. This is an acute, high dose, septic infection model, intended to examine innate host defenses. A major defense mechanism in larva is hemocytes, which are phagocytes analogous to neutrophils in humans (Wojda et al., 2020). Since a hallmark of human *Campylobacter* infections is gut inflammation (Omole et al., 2024), and neutrophil infiltration into tissues is a major inflammatory marker, bacteria-phagocyte interactions are important to examine. However, how well a disseminated infection (bacteria are injected into the larval body cavity) predicts the outcome of a human or chicken gut infection remains to be seen, as tissues are sterile yet the gut is a microbiologically dense, competitive environment. The infective dose of *C. jejuni* in humans is approximately 1000 bacteria, while the larva are typically given 10^5^ to 10^7^ CFU. Given that larva body weights are 150–300 mg and a typical adult human body weight is 62 kg, relatively that is more than a million-fold higher dose. A generic description of the larva killing assay (see **Table 1** for more details) is: *Campylobacter* is grown under microaerobic conditions on plates (Columbia blood or MHA) or broth (Brucella or MHB) and suspended in PBS, with bacterial concentration approximated using OD (adjusted to 0.1 or 1.0). Larva maintained at lower temperatures (10 to 17°C) and starved for 2-8d are injected with 10 μL of the bacterial suspension, typically near the right foreleg, then transferred to 37°C and observed for darkening (the immune melanization response) and death, which is often rapid (24 hrs or less). Groups of ten larva are used for different conditions or strains, often in triplicate (so total n=30 for each condition). In Champion’s foundational study (Champion et al., 2010), three strains (11168H, G1, and 81-176) displayed equivalent killing (~70% at 24 hrs and 37°C) when grown on Columbia blood agar plates. Interestingly, strain 11168H grown in MHB killed all infected larva by 24 hrs (data from other two strains not shown). Perhaps the physiological state of the bacteria is different (such as flagella being used more in liquid versus the plate), or the suspension method from plates may expose the bacteria to more oxygen than when from broth. The larva are in a stressed state, since they have been starved for up to a week, and experienced a major temperature shift just after infection (from 15°C to 37°C). However, uninfected controls were included and none died in the 24 hr observation period. The authors of this publication chose to display survival of the larva at only one timepoint with column graphs, each column representing one group. This choice has been replicated often in the field as the dominant visualization of the killing assay. Note, with other animal challenge studies, the standard choice is Kaplan-Meier survival curves. The *C. jejuni* strain most commonly used for infection of *G. mellonella* is NCTC 11168 (and its hypermotile derivative, 11168H), see **Table 1**. While 11168 is referred to as the type strain in some publications (Pascoe et al., 2019; Whelan et al., 2019), the ATCC lists the *Cj* subsp. *jejuni* type strain as the bovine isolate CIP 702, also known as NCTC 11351 or ATCC 33560 (Campylobacter jejuni subsp. jejuni (Jones et al.) Steele and Owen, 2016).

**Table 1:** Summary of infection assay methods.

Reference	C. *jejuni* Strains	Growth	Source	Housing	Inoculation	Incubation	Survival
(Champion et al., 2010)	11168H 81-176 G1	CAB with HB 5%-9% or Skirrow Suppl. MHB at MA1/shaking	Livefoods Direct Ltd (UK)	Wood chips, 15°C	10 μL injection in PBS, OD_590_=1.0^A^, 10^6^ CFU into right foreleg	37°C, 24 hrs, assume aerobic^F^	Plate Grown 81-176 – 43% 11168H – 36% G1 – ~25% Broth Grown 11168H - 0%
(Senior et al., 2011)	11168H 11168-O 81116 81-176	MHB with CPK/shaking	Livefoods Direct Ltd (UK)	Wood chips, 15°C	10 μL injection in PBS, OD_590_=1.0, 10^6^ CFU^B^ into right foreleg	37°C, 24 hrs, aerobic	11168H – 0% 11168-O – 27% 81116 – 90% 81-176 – 90%
(Gundogdu et al., 2011)	11168H	CAB with HB 7% and CSS, BB at MA1/shaking	Livefoods Direct Ltd (UK)	Wood chips, 16°C	10 μL injection in PBS, OD_600_=0.1^B^, 10^6^ CFU into right foreleg	37°C, 24 hrs, aerobic	11168H – ~45%
(Elmi et al., 2012)	11168H	CAB + HB 7% and CSS, BB at MA1/shaking	Livefoods Direct Ltd (UK)	Wood chips, 16°C	10 μL injection in PBS, OD_600_=0.1^B^, 10^6^ CFU into right foreleg	37°C, 24 hrs, aerobic	11168H – ~70% OMVs from: 11168H – 70%
(Van Alphen et al., 2014)	11168H 81-176	MHA, MHB at MA1/shaking	Bug Order Inc. (CA)	Wood chips, 4°C	5 μL injection in MgSO_4_, OD_550_=1.0, 10^9^ CFU^B^ in left hindmost pro-leg	37°C, 24 hrs, aerobic	11168H – ~12% 81-176 – ~30%
(Humphrey et al., 2015)	11168H M1 13126 12662 DBM1	CAB with DHB (5%), MHB at 5% O_2_/ 12% CO_2_/ 3% H_2_/ 80% N_2_	Livefoods Direct Ltd (UK)	Not specified - Ref Gundogdu 2011	10 μL injection in PBS, 10^6^ CFU^B^ into haemocoel (unspecified)	37°C, 48 hrs, aerobic	11168H – 67% M1 – 88% 13126 – 63% 12662 – 95% DBM1 – 78%
(Gundogdu et al., 2015)	11168H	CAB with HB 7% and CSS, BB at MA1/shaking	Livefoods Direct Ltd (UK)	Wood chips, 16°C	10 μL injection in PBS, OD_600_=0.1^B^, 10^6^ CFU into right foreleg	37°C, 24 hrs, aerobic	11168H – ~45%
(Handley et al., 2015)	11168	BA, Blood Agar Base Agar with Skirrows Suppl., BB at MA1/shaking	Livefoods Direct Ltd (UK)	Not described	10 μL injection in PBS, OD_600_=0.1^B^, 10^6^ CFU into right foremost pro-leg.	37°C, 24 hrs, aerobic	11168 – ~20%
(Reuter et al., 2015)	11168	Blood Agar Base Agar with Skirrows Suppl. at MA1	Livefoods Direct Ltd (UK)	Not Specified	10 μL injection in PBS, OD_600_=1.0, 10^6^ CFU^B^ into right foremost pro-leg	37°C, 24 hrs, aerobic	11168 – ~30%
(Jowiya et al., 2015)	11168H 81-176	MHB, BA, CAB with DHB 7% with CPK	Cornish Crispa Co. (UK)	Wood chips, 12°C	10 μL injection in PBS, 10^6^ CFU^B^ into right forelegs	Unspecified temp for 24 hrs^G^, aerobic	11168H – ~90%^H^ 81-176 – ~80%
(Gundogdu et al., 2016)	11168H 81-176 81116 M1	CAB with HB 7% and CSS, BB at MA1/shaking	Livefoods Direct Ltd (UK)	Wood chips, 16°C	10 μL injection in PBS, OD_600_=0.1^B^, 10^6^ CFU into right foreleg	37°C, 24 hrs, aerobic	11168H – ~45% 81-176 – ~65% 81116 – ~50% M1 – ~50%
(Askoura et al., 2016)	11168	Biphasic MHA/MHB at 8% O_2_, 5% CO_2_, 4% H_2_, 83% N_2_	Gecko Gurl (CA)	10-15°C, wood chips assumed	10 μL injection in PBS, 10^5^-10^7^ CFU^A^ into left hindmost pro-leg	25°C for 144 hrs on wood chips	11168 10^6^ CFU: 24 hrs – ~80% 48 hrs – ~60% 72 hrs – ~40% 120 hrs – ~20% 144 hrs – ~0%
(Askoura and Stintzi, 2017)	Selected strain(s)	Biphasic MHA/MHB at 8% O_2_, 5% CO_2_, 4% H_2_, 83% N_2_	“trusted vendor”	Wood chips, 10-15°C	10 μL injection in PBS, 10^5^-10^7^ CFU^A^ into left hindmost pro-leg	Room temp, aerobic	N/A
(Tang et al., 2017)	11168-GS^I^ 111680-O	Sheep Blood Agar + Skirrows Suppl., Biphasic MHB/MHA at 7.5% CO_2_/ 7.5% O_2_/ 5% N_2_/ remainder not stated	Livefoods Direct Ltd (UK)	Wood chips, 15°C	10 μL injection in PBS, OD_600_=0.6, 10^6^ CFU^B^ into right foremost pro-leg	37°C and 42°C for 24 hrs, aerobic	37°C: 11168-GS – 83% 11168-O – ~70% 42°C:^L^ 11168-GS – 23% 11168-O – ~65%
(Mehat et al., 2018)	81116 M1	MHA + Sheep blood 5%, MHB with CPK	Livefoods Direct Ltd (UK)	Wood chips, 16°C	10 μL injection in PBS OD_590_=1.0^B^, 10^6^ CFU into right foremost pro-leg	37°C for 24 hrs, aerobic	81116 – ~10% M1 – ~5%
(Elmi et al., 2018)	11168H^C^	BB + 0.1 or 0.2% Na taurocholate (ST), MA1, 37°C^D^	Livefoods Direct Ltd (UK)	Wood chips, 16°C	10 μL injection in PBS, 5 μg of OMVs (no bacteria injected), right foreleg	37°C for 72 hrs, aerobic	OMVs from: 11168H – 55% + 0.1% ST - ~35% + 0.1% ST - ~30%
(Brunner et al., 2018)	11168H	Blood agar no.2 with 0.5% yeast extract and DHB (5%) with CPK	UK Waxworms Ltd. (UK)	No specified	10 μL injection in PBS, 10^7^ CFU^B^ into second foreleg	37°C for 120 hrs, aerobic	11168H: 24 hrs – ~90% 48 hrs – ~80% 72 hrs – ~60% 96 hrs – ~50% 120 hrs – ~35%
(Liaw et al., 2019)	488 81-176	CAB with HB (7%) and Skirrows Suppl. at MA1	Livefoods Direct Ltd (UK)	Not Specified	10 μL injection in PBS OD_600_=0.1^B^, no CFU approximation, into right foremost foreleg	37°C for 120 hrs, aerobic	488: 24 hrs – ~90% 120 hrs – ~50% 81-176: 24 hrs – ~100% 120 hrs – ~75%
(Whelan et al., 2019)	11168 81-176	MHB with CPK	Livefoods Direct Ltd (UK)	Wood chips, 15°C	20 μL injection in PBS, OD_600_=1.0, 10^1^-10^8^ CFU^A^ into right hindmost pro-leg	37°C for 72 hrs, aerobic	11168 10^6^ CFU: 24 hrs – ~90% 48 hrs – ~90%
(Bojanic et al., 2020)	*Campylobacter spp.* (34 strains *- C. jejuni*)	Columbia blood agar at 10% CO_2_/ 3% O_2_/ 82% N_2_/ 5% H_2_	Biosuppliers (NZ)	Woodchips and honeycomb, 25°C	10 μL injection in PBS, OD_590_=.05, 10^4^, 10^6^, and 10^8^ CFU^A^ into left hindmost pro-leg	37°C for 288 hrs, microaerobic^J^	Aggregated C. jejuni: 24 hrs – ~95% 72 hrs – ~73% 120 hrs – ~60% 240 hrs – ~50%
(Barnawi et al., 2020)	11168	MHA, MHB at MA1	Livefoods Direct Ltd (UK)	Not Specified	10 μL injection in PBS, OD_600_ = 1.0, 10^6^ CFU^B^ into hindmost pro-leg	32 - 37°C for 144 hrs, aerobic	11168 10^6^ CFU at 37 °C: 24 hrs – ~50% 72 hrs – ~20% 120 hrs – ~0% 11168 10^7^ CFU at 32 °C 24 hrs – ~100% 72 hrs – ~95% 120 hrs – ~90%
(Emery et al., 2021)	BAA-224	MHB or MHA with Anaerocult P^K^	Livefoods Direct Ltd (UK)	On SD, 28-30°C	10 μL injection in PBS, 1 & 3x10^6^ CFU^A^ into left hindmost pro-leg and orally	30°C for 72 hrs, aerobic	BAA-224 10^6^ Force Fed: SD – ~70% BAA-224 10^6^ injected: SD – ~85%
(Benoit and Maier, 2022)	11168 81-176	BA with defibrinated sheep blood 10% at 10% CO_2_/ 4% O_2_/ 86% N_2_	Bug Company (US)	Wood chips, 4°C	5 μL injection in PBS, OD_600_ = 1.0, 2.7x10^7^ CFU^A^ into left hindmost pro-leg	37°C for 144 hrs, aerobic	11168: 24 hrs – ~50% 72 hrs – ~40% 120 hrs – ~20% 81-176: 24 hrs – ~60% 72 hrs – ~40% 120 hrs – ~20%
(Myles et al., 2024)	11168	Tryptic soy agar with sheep blood 5%, Tryptic soy broth, BB at MA1/shaking	Recorp Inc. (US)	Wood chips, 15°C	10 μL injection in 0.85% saline, OD_600_=3.0, 5x10^9^ CFU^A^, into right foremost foreleg	37°C for 144 hrs, aerobic	11168: 25 hrs – ~25% 42 hrs – ~12%
(Gomes et al., 2024)	ATCC 33291^E^	Not specified	Not specified	Glass, 28°C, fed (but food not described)	5 μL injection in PBS, approximated 5x10^6^ CFU^B^, into right hindmost pro-leg	37°C for 192 hrs (8 days), aerobic	ATCC 33291: 48 hrs – ~100% 72 hrs – ~80% 192 hrs – ~80%

BA, Brucella Agar; BB, Brucella Broth; BHIA, Brain Heart Infusion Agar; CAB, Columbia Agar Base; CSS, Campy Selective Supplement; DHB, Defibrinated Horse Blood; HB, Horse Blood; MHA, Mueller-Hinton Agar; MHB, Mueller-Hinton Broth. MA1 = microaerobic one (5% O_2_, 10% CO_2_, 85% N_2_), CPK = 5% O_2_, 10% CO_2_, approximately generated by CampyPak (Becton Dickinson). SD (Standard Diet) = corn meal (90 g), wheat flour (50 g), dried milk (50 g), beeswax (50 g), honey (50 g), glycerol (50 g), dry yeast (22 g), and distilled water (50 g).

^A^Doses confirmed by serial dilution and plating onto CBA (data not shown). Stated as “approximately 10^6^ CFU” per worm.

^B^Doses rely on absorbance values and are not stated to be confirmed by plating.

^C^11168H was the strain that OMVs were derived from.

^D^Culture conditions under which OMVs were generated.

^E^Also tested 18 *C. coli* strains, data not shown here.

^F^Atmospheric aerobic conditions are assumed for the worms, although in most studies that is not stated.

^G^Worm incubation conditions not given, but since authors refer to Champion 2010, it is likely 37°C.

^H^Survival of worms injected with 11168H exposed to amylase was 15% and amylase-exposed 81-176 was 10%.

^I^11168-GS is the isolate that was genomically sequenced, while 11168-O is the initial clinical isolate (Gaynor, EC et al.,*J Bacteriol* 2004).

^J^Microaerobic was 10% CO_2_/ 3% O_2_/ 82% N_2_/ 5% H_2_.

^K^Merck product literature states that the Anaerocult P system is supposed to generate environments with no oxygen and some CO_2_.

^L^The bacteria were cultured at 42°C. After injection, the worms were incubated at 37°C.

## Capsule

Capsular polysaccharide structures exhibit unusually wide variety between *C. jejuni* strains, and the serotypes are often due to phase-variable modifications such as ethanolamine, O-methyl, and other modifications (Van Alphen et al., 2014). Gene knockout mutants lacking transferase activity for adding the O-methyl phosphoramidate (MeOPN) modification to the capsule exhibited the same lethality (~70-80% killing at 24 hrs) as the respective wt strains (81–176 and 11168H, which were also statistically indistinguishable), while the acapsular mutant (ΔkpsM in 81-176) had only 20% killing. However, mutants which cannot synthesize the MeOPN (knockout of cj1415 in 81–176 or cj1416 in 11168H) did have lower (~40%) larva killing. The authors proposed that since transferase mutants still contain MeOPN modified sugars while the biosynthetic mutants do not, this may account for differences. However, pure MeOPN monomers or purified capsule displayed no larva killing. Alternatively, the biosynthetic mutants may contain less capsule overall. Note that p-values were given for larva killing assays, but the statistical test was not specified, yet it was likely an unpaired t-test, as that was used in other assays. As this test assumes equal variance between groups and normal distribution of data, it may not be the appropriate test for this data. In comparison, Champion et al. (Champion et al., 2010) had 100% killing by 11168H and ~10% by Δ1416, while van Alphen et al. (Van Alphen et al., 2014) had 70% killing by 11168H and 30% by Δ1416 mutant. The van Alphen study had a different source of larva (Bug Order Inc, Alberta vs Livefood, UK for Champion), held larva at 4 °C for up to a week before infection, had 5 μL injections, and uniquely suspended bacteria in 10 mM MgSO_4_ (pH not given) rather than PBS. Champion determined doses by plating and estimated 10^6^ bacteria per animal, while van Alphen estimated doses at 5 x 10^6^ per larva. Methodological differences such as these limit direct comparisons across studies. In a later study also focused on the capsule (Myles et al., 2024), four gene knockout mutants disrupted in adding hexoses to the capsule structures displayed equivalent killing in larva to the wt 11168 (~80% dead at 17hrs). However, the dose was extremely high (5x10^7^ per worm), killing was abnormally rapid, the saline negative control larva had ~20% death, and the acapsular mutant *ΔkpsM* also had equivalent killing. This massive dose may have simply overwhelmed the host defenses or killed them via chemical toxicity (i.e. LOS); note, a heat-killed bacterial control examining that possibility was not reported.

## Transcriptional regulators

Five different studies have examined transcriptional regulators related to *Campylobacter* virulence with larva. Knockout of the cj1556 regulator gene (*rrpB*) reduced larva killing from ~55% with the wt 11168H to ~35% with the mutant (Gundogdu et al., 2011). While it was stated that the p-value was less than 0.05 (actual value not given), the Student’s t-test was used (limitations same as above). Also, the methods state that survival was recorded in 24hr intervals, but the total observation time was not given. Knockout of the related regulator gene *rrpA* reduced killing at 24hrs from ~55% with 11168H to ~25% with the mutant (Gundogdu et al., 2015). Surprisingly, the double mutant was not statistically different from wt in killing (the authors suggest compensatory regulation but don’t have transcriptional data). Also, knockouts in the oxidative defense genes *katA*, *ahpC*, and *sodB* had apparently reduced killing, but not statistically significant. In a related study, *rrpA* deletion in 81–176 also had reduced killing, but deletion in strain 81116 was not significantly different from the wt (Gundogdu et al., 2016), suggesting the genetic background is important. While deletion of the *perR* (cj0332) regulator, which represses expression of several oxidative stress genes, increased aerotolerance, it did not affect killing of larva (Handley et al., 2015). Deletion of the iron uptake regulator *fur* in strain 11168 also reduced larva killing significantly (LD_50_ increased from 3.1 x 10^5^ for wt to 2.6 x 10^6^ for the mutant) (Askoura et al., 2016).

## Outer membrane vesicles

Proteins in outer membrane vesicles (OMVs) from *C. jejuni* 11168H were identified (Elmi et al., 2012). Injection of an OMV preparation containing 5 μg of total protein killed larva similarly (~30%) as a dose of live bacteria (~10^6^ CFU). A lower dose (0.5 μg) killed less and heat-treated OMVs had no killing at any dose, suggesting the killing is not due to LOS content. In a second study from the same group (Elmi et al., 2018), the same 5 μg protein dose of 11168H OMVs killed more larva (45%), and when the OMVs came from bacteria grown with taurocholate (shown to have increased protease activities), killed ~70%. Given the same conditions stated in the methods sections of those two studies, the reason for the difference is unclear.

## Other virulence factors

Deletion of *capC*, a virulence factor of unknown function found in the outer membrane, reduced killing of larva by both strain 8116 and M1 (Mehat et al., 2018). A type six secretion system (T6SS), which are phage-related structures that inject effoctor proteins into neighboring cells, may play a role in *C. jejuni* virulence. Deletion of a critical factor (*tssD*) in T6SS from the genome of clinical strain 488 reduced killing of larva (Liaw et al., 2019). Additionally, a clinical isolate lacking the T6SS gene cluster (strain 81-176) exhibited lower killing than the wt 488, although not statistically significant. The role of T6SS during human infections remains unclear, as a screen in 366 isolates showed the gene cluster in only 4.7% of the strains, none of them from human sources (Siddiqui et al., 2015). Isolates resistant to nalidixic acid or ciprofloxacin (three strains for each antibiotic) were generated *in vitro*, and displayed increased killing (i.e., LD_50_ for wt 11168 at 24 hrs of 10x7.59 ± 0.02 CFU/larva while resistant strains had LD_50_ values ranging from 10x6.60 to 6.01) (Whelan et al., 2019). *In vitro* growth in MHB was reported to be the same, although instead of growth curves, the OD600 was shown at one time point only. Interestingly, the mutants had decreased motility, increased biofilm formation, and increased adherence to HT29 epithelial cells. This suggests these properties may be related to larva killing. A related study reported no fitness cost for fluoroquinolone resistance during a competition assay for chicken colonization (Zhang et al., 2003) while another reported resistant *C. jejuni* strains were less competitive in food and chicken gut (Zeitouni and Kempf, 2011). These conflicting results suggest that genetic background or other factors may control how mutations in the target gyrase gene affect bacterial fitness.

## Virulence signals

Growth of two strains of *C. jejuni* (11168H and 81-176) on MHA in the presence of pancreatic amylase dramatically increased larva killing at 24hrs, from 10-20% without treatment to 90% with (Jowiya et al., 2015). Treated bacteria generate an extracellular dextran, have enhanced biofilm formation, and have increased adhesion and invasion of Caco-2 cells. Controls of larva injected with amylase or dextran were not reported.

## Temperature

While *Campylobacter* causes gut inflammation in mammals (37°C body temperature), chickens colonized with the bacteria have few overt effects (42°C body temperature), thus temperature is investigated as a potentially important factor in virulence regulation. Proteomic analysis was used to identify proteins differentially regulated between these two temperatures (Tang et al., 2017). Strain 11168 was more lethal to larva when coming from culture at 42°C (~80% killing at 24 hrs) compared to 37°C (~20% killing). This is interesting given the lower pathogenicity of *Campylobacter* in chickens, suggesting factors other than temperature are important. Strain 11168 displays much less larva killing at 32°C compared to 37°C (Barnawi et al., 2020). For comparison, the minimal growth temperature determined for strains 104 and 33560 was 32 and 31°C, respectively (Hazeleger et al., 1998), while the optimal growth range for *Galleria* is 25-33°C. This study (Barnawi et al., 2020) also interestingly suggested that the ability to tolerate zinc may be important in larva, as a mutant which continues expression of zinc exporter *czcD* (normally repressed at 32°C) kills more larva than the wild-type at 32°C. The combination of the nickel chelator DMG (dimethylglyoxime) with copper was found to kill *C. jejuni in vitro*, with an MBC value versus 11168 of 8 μM for copper in the presence of 4 mM DMG in MHB (Benoit and Maier, 2022). This effect may be restricted to *Campylobacter*, as the growth of four related genera (*Klebsiella*, *Escherichia*, *Salmonella*, and *Acinetobacter*) were unaffected by the combination (DMG concentrations up to 5 mM and copper up to 250 μM). Additionally, larva injected with a DMG/copper (10 mM/1mM) combination two hours before bacterial injection had decreased and delayed killing with both 1168 and 81-176. However, no copper only or DMG only control was included. Inclusion of DMG/copper in drinking water also decreased colonization of chickens by 11168. Further investigation into this potential new therapeutic is warranted.

## Species and strain comparisons

The ease of the *Galleria* model allows virulence comparisons across many different strains or species quickly and with relatively high numbers of animals. Sixty-seven *C. jejuni* isolates from humans, chickens, and other animals were screened for larva killing (Senior et al., 2011). While data for individual strains was not shown, larva survival was shown when infected with strains placed into six MLST groupings, although only one pairwise comparison (ST21, least killing, vs ST257, most killing) was statistically different. However, screening of six strains of *C. jejuni* in larva revealed the highest killing rate (~35% at 48hrs) by strain 13126, a poultry isolate, also type ST21 (Humphrey et al., 2015). Given the genetic diversity even within MLST groupings, this is not surprising. Four clinical isolates of *C. concisus* had less larva killing than *C. jejuni* 1168H (Brunner et al., 2018). In a more extensive study, 22 strains of *C. upsaliensis* and 13 strains of *C. helveticus* (both mainly isolated from cats and dogs) had less killing of larva that 34 C. *jejuni* strains (Bojanić et al., 2020). Incubation of infected larva was uniquely done under microaerobic conditions, where about 10% of uninfected control larva died after 10d. Three of 18 strains of *C. coli* killed fewer larva than the *C. jejuni* reference strain, while the other 15 strains caused higher mortality. (Gomes et al., 2024) However, the reference strain was clinical isolate ATCC 33291, not the commonly used 11168, so how it compares to other studies is unclear.

## Summary and future directions

The most prolific authors in this field have been Nick Dorrell (7 publications, 6 as senior author), Brendan Wren (9 publications), Abdi Elmi and Ozan Gundogdu (6 publications each), and Mona Bajaj-Elliott and Arnould van Vliet (4 publications each). The dominant strategy of high dose, acute, disseminated infection in *Galleria* has helped reveal the role of multiple virulence factors, compare virulence across strains/species, and begin to understand the relationship of temperature and infection. When examined, the results of the larva killing assays generally line up with those from chick colonization or cultured cell invasion. The lack of killed bacterial cell controls is a limitation in most studies, given the rather high lethality (~50% by day 4) of heat-killed cells (Bojanić et al., 2020). Since high bacterial doses are injected into larva, some of the lethality may be coming from bacterial products such as LOS or cell wall materials, which is a confounding factor when comparing across studies. Another potential confounding factor is that larva sizes are not described in these studies and is probably varying, and larva weight has been shown to affect lethal dose with *Staphylococcus* (Hesketh-Best et al., 2021). A third issue is that the infected larva are incubated under normal atmospheric oxygen levels, and while that may be appropriate to model extra-intestinal tissue infections in humans, *Campylobacter* by far remains in the anaerobic gut in both humans and chickens. Further, *Campylobacter* was much more lethal to larva incubated under microaerobic compared to aerobic conditions (Bojanić et al., 2020), which deserves more study. In most protocols, larva are held at 10-15°C before bacterial injection, then at 37°C after. How this affects larva immunocompetency is unclear, but temperature shifts of larva can increase hemocyte density, expression of immune genes, and resistance to killing by *Candida* (Mowlds and Kavanagh, 2008). However, starvation (for 7d) can lower hemocyte count and increase susceptibility of larva to *Candida* (Banville et al., 2012). One conflict in the current literature is whether larva can be incubated at 42°C, as one study reports such data (Senior et al., 2011) (however, study does not show any control larva data at 42°C) while another reports that larva cannot survive at that temperature (Champion et al., 2010). In our hands and others (Vertyporokh et al., 2015; Liu et al., 2019), the larva cannot survive at 42°C, so perhaps larva from different sources have varying temperature tolerances. In future studies, the oral route of inoculation, called force feeding, may be more revealing than the injection route. Oral would allow for examining *Campylobacter* interactions with the gut microbiome and with food, determining crossing of the gut barrier and invasion into tissues, and initial analysis of potential probiotics. In one study that included force feeding and body injection (Emery et al., 2021), the oral dose had higher lethality (25% killing at 24 hrs) than the injection method (10% killing). However, comparison with other studies is unclear, as the larva incubation temperature was lower (30°C), the BAA-224 *Campylobacter* strain was unique, and larva were fed previously, while commonly they are starved. Applying oral infection studies would necessitate a more thorough determination of the larva gut microbiome and how it is affected by experimental manipulations, like incubation at lowered temperature or starvation. Finally, direct comparisons of strains in larva with other models, such as chickens (Humphrey et al., 2015), will help validate the *Galleria* system.
